# Neoplastic diseases in the domestic ferret (*Mustela putorius furo*) in Italy: classification and tissue distribution of 856 cases (2000–2010)

**DOI:** 10.1186/s12917-016-0901-7

**Published:** 2016-12-05

**Authors:** Giancarlo Avallone, Annalisa Forlani, Marco Tecilla, Elena Riccardi, Sara Belluco, Sara Francesca Santagostino, Guido Grilli, Kiumars Khadivi, Paola Roccabianca

**Affiliations:** 1 Department of Veterinary Medical Sciences (DIMEVET), University of Bologna, Ozzano dell’Emilia, Italy; 2 IDEXX Laboratories Ltd. Grange House, Sandbeck Way, Wetherby, West Yorkshire, LS22 7DN UK; 3 Department of Veterinary Medicine, University of Milan, Via Celoria 10, 20100 Milan, Italy; 4 Equipe recherche UPSP ICE 2011-03-101: Oncology, and Laboratoire de Pathologie et Morphologie Clinique, Vetagro-sup, France; 5 Laboratory of Comparative Pathology, Memorial Sloan Kettering Cancer Center, 1275 York Av, Box 270, New York, USA; 6 Private practicioner, Milan, Italy

**Keywords:** Ferret, Tumours, Neoplastic diseases

## Abstract

**Background:**

The aim of this study was to describe the prevalence and tissue distribution of neoplasms in Italian ferrets, compared to the epidemiological data previously reported in USA and Japan.

**Methods:**

Signalment and diagnoses of pathological submissions received between 2000 and 2010 were searched; cases with the diagnosis of neoplasm were selected and original sections reviewed to confirm the diagnosis.

**Results:**

Nine-hundred and ten samples were retrieved, 690 of which included at least one tumour for a total of 856 tumours. Ferrets with multiple neoplasms were 134 (19.4%). Median age was 5 years, and F/M ratio was 0.99. Endocrine neoplasms were the most common. Other frequent tumours were cutaneous mast cell tumours, sebaceous tumours, and lymphomas. Cutaneous squamous cell carcinomas (SCC) were consistently associated with sebaceous tumours. Twenty-four abdominal spindle cell tumours with an undefined origin were observed. Lymphomas and islet cell tumours had a lower incidence compared with previous extra-European studies.

**Discussion:**

Epidemiological information on ferret tumours derives from extra-European countries, mostly USA and Japan. In these countries similar distributions with minor discrepancies have been reported. Compared to previous reports, adrenal tumours were more frequent than pancreatic islet cell neoplasms, and a higher number of mesenchymal neoplasms arising from the adrenal capsule was noted. An unusual association between SCC and sebaceous gland neoplasms and a high number of intrabdominal spindle cell neoplasms with unclear primary origin were noted and grants further investigation.

**Conclusions:**

The tissue distribution of tumours recorded in this study paralleled previous findings in ferrets from USA and Japan. Some differences have been noted in the frequency of lymphoma, adrenal mesenchymal tumours and cutaneous tumours. Some tumours that are among the most common in other species seem to be uncommon in ferrets and are characterized by distinctive predilection sites.

## Background

The ferret (*Mustela putorius furo*) is a popular non-conventional companion animal for which a prevalence of neoplastic diseases ranging from 5.2 to 12% has been described [1–4].

Tumours of the endocrine system are the most common neoplasms in ferrets with a majority of pancreatic islet and adrenal tumours [1–4]. Pancreatic islet beta cell tumours represent the most frequent neoplasm and almost the totality of primary pancreatic neoplasms in ferrets [1–5]. Genetic and nutritional components have been postulated to predispose ferrets to the onset of this tumour type in the United States [5]. Adrenal tumours have been reported as the second most frequent endocrine disease both in the United States and Japan [1–4]. The pathogenesis leading to adrenal gland disease is still debated; however, early gonadectomy combined with an artificially prolonged photoperiod experienced by indoor pet ferrets, along with a possible genetic component, seem to predispose to adrenal tumours [6].

The second most common affected systems are the hemolymphatic and the integumentary system, with similar frequencies [1–4]. Hemolymphatic neoplasms have a slightly higher frequency in ferrets from North America [1], while the contrary occurs for integumentary neoplasms in ferrets from Japan [3]. The epidemiological similarities of neoplastic disease between Japanese and U.S. ferrets could be explained by the fact that most Japanese pet ferrets are imported from North America, and their husbandry, including diet, is comparable [3].

To the best of our knowledge, there are no epidemiologic reports with reference to domestic ferret neoplastic diseases in Europe and, specifically, in Italy. This represents an 11-year retrospective study investigating tumour types and their prevalence in ferrets from Northern Italy.

## Methods

The electronic archives of the exotic and non-conventional companion animal diagnostic service were searched from its establishment in 2000 till 2010 covering a 11-year period. Records of histopathological samples from ferrets (biopsies and necropsies) including at least one neoplastic lesion were retrieved, and data regarding gender, age, breed, site of the lesions and histological diagnoses were collected. In Italy, early gonadectomy is frequent in ferrets; however the neutering status was inconsistently included, therefore, gender was categorized into male (61 ferrets), female (76 ferrets), neutered males (252 ferrets), neutered females (238 ferrets) and unknown (59 ferrets).

Tumour types were classified according to the published references or the pertinent fascicles of the World Health Organization “International Histological Classification of Tumours of Domestic Animals” [7].

Neoplasms were grouped by affected organ system (e.g. skin and subcutis, gastrointestinal tract, etc.) with the exclusion of lymphoid neoplasms that were grouped independently from the organ or tissue of origin. Cases of neoplastic diseases with low frequency were grouped in a set named “miscellaneous”. Neoplasms developing in the same ferret but with different tissue of origin and different malignancy were recorded as independent neoplasms using the same identification number for the ferret. Hyperplastic lesions were excluded. The diagnoses were reviewed independently by 4 board certified pathologists. Differences between the diagnoses were discussed jointly to achieve an agreement.

## Results

The number of pathological specimens collected from ferrets during an 11-year period was 910. Of these, 690 samples contained at least one neoplastic lesion. Multiple neoplasms occurred in 134 cases (19.4%): 111 ferrets were diagnosed with two tumours, 16 with three, 5 with four, and 2 with five different tumours. Consequently, a total of 856 tumours was included in this study. The tissue distribution of all neoplasms excluding lymphoid tumours is summarized in Table 1. The tissue distribution of lymphoid neoplasm is summarized in Table 2.

**Table 1:** tissue distribution and specific diagnosis of tumors in ferrets

Organ system	Differentiation/site	Diagnosis	N° of cases
Adrenal gland	Cortical	Cortical carcinoma	228
		Cortical adenoma	61
	Medullary	Pheochromocytoma	11
		Neuroblastoma	2
	Other	Leiomyoma	5
		Spindle cell sarcoma	3
		Leiomyosarcoma	2
		Malignant neoplasia NOS	1
Pancreas	Insular	Islet cell tumor	190
	Exocrine	Exocrine adenocarcinoma	6
		Exocrine adenomas	4
Skin and subcutis	Mastocytic	Mast cell tumor	68
	Sebaceous	Sebaceous epithelioma	22
		Sebaceous adenoma	16
	Apocrine	Apocrine adenocarcinoma	10
		Apocrine adenoma	8
	Squamous	Squamous cell carcinoma	9
	Vascular	Hemangioma	8
		Angiosarcoma	1
	Other	Fibroma	3
		Leiomyoma	3
		Basal cell carcinoma	2
		Trichoblastoma	2
		Leiomyosarcoma	1
		Rhabdomyosarcoma	1
		Infundibular keratinizing acanthoma	1
		Lipoma	1
Skeletal system		Chordoma	24
		Osteosarcoma	2
Abdominal cavity		Spindle cell tumor with polygonal cells	22
		Leiomyosarcoma	1
		Hemangioma	1
Liver	Cholangiocellular	Cholangiocellular carcinoma	6
	Vascular	Hemangiosarcoma	4
		Hemangioma	2
	Hepatocellular	Hepatocellular carcinoma	3
		Hepatocellular adenoma	2
	Round cell tumor	Undifferentiated NOS	3
Digestive tract	Oral cavity	Squamous cell carcinoma	3
		Fibromatous epulis	1
		Sarcoma NOS	1
	Stomach	Gastric carcinoma	3
	Intestine	Adenocarcinoma	6
		Leiomyoma	1
Genital system	Ovary	Gonadostromal tumor	3
		Carcinoma	1
	Uterus	Adenocarcinoma	2
		Leiomyoma	1
	Vagina	Papilloma	1
	Testicle	Seminoma	3
		Gonadostromal tumor	2
	Prostate	Adenocarcinoma	1
Miscellaneous	Lymph node	Metastatic neoplasm	4
		Hemangioma	1
	Spleen	RCT NOS	3
		Myelolipoma	1
	Kidney	Sarcoma NOS	1
	Lung	Adenocarcinoma	1
	Mediastinum	Hemangioma	1
	Thoracic cavity	Sarcoma NOS	1
	Salivary gland	Adenoma	1
	Lacrimal gland	Adenoma	1

**Table 2:** Tissue distribution of hemolymphatic tumors in ferrets

Differentiation	Distribution	N° of cases
Lymphoma	Nodal	16
	Splenic	14
	Gastrointestinal	13
	Multicentric	8
	Cutaneous	5
	Hepatic	5
	Hepatosplenic	5
	Pancreatic	1
	Renal	1
	Intra-abdominal NOS	2
Plasma cell tumor	Splenic	2
	Nodal	1
	Multicentric	1

**Fig. 1:**
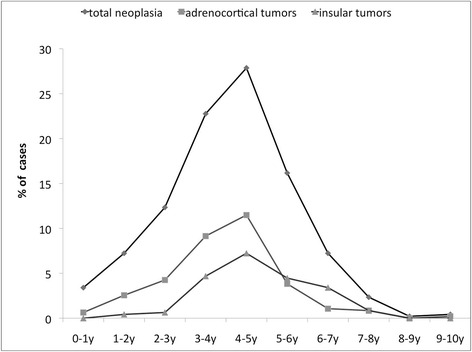
Histogram representing age distribution for all neoplastic lesions (*line with squares*) and for each of the most represented neoplastic categories: adrenocortical (*line with diamonds*), insular (*line with triangles*) and hemolymphatic (*lines with circles*) tumors in ferrets. The number of cases is expressed as percentage of the total cases for which the age was indicated

Specifically, of the 856 tumours, 313 adrenal tumours were collected, along with 200 pancreatic, 156 cutaneous and subcutaneous, 74 lymphoid, 26 skeletal, 24 intra-abdominal, 20 hepatic, 15 digestive tract, and 14 genital tumours. Fifteen cases were grouped under the “miscellaneous” category. Age was recorded in 459 cases. The mean age of affected ferrets was 4.5 years, and the median was 5, with a range from 7 months to 10 years. Age distribution in all affected ferrets and in ferrets with adrenocortical, insular and hemolymphatic tumours, is summarized in Fig. 1. Gender was recorded in 641 cases (321 males and 320 females). The F/M ratio was 0.99. Endocrine neoplasms were the most common (57.0%), followed by cutaneous/subcutaneous (18.2%) and lymphoid tumours (8.6%).

### Adrenal gland

**Fig. 2:**
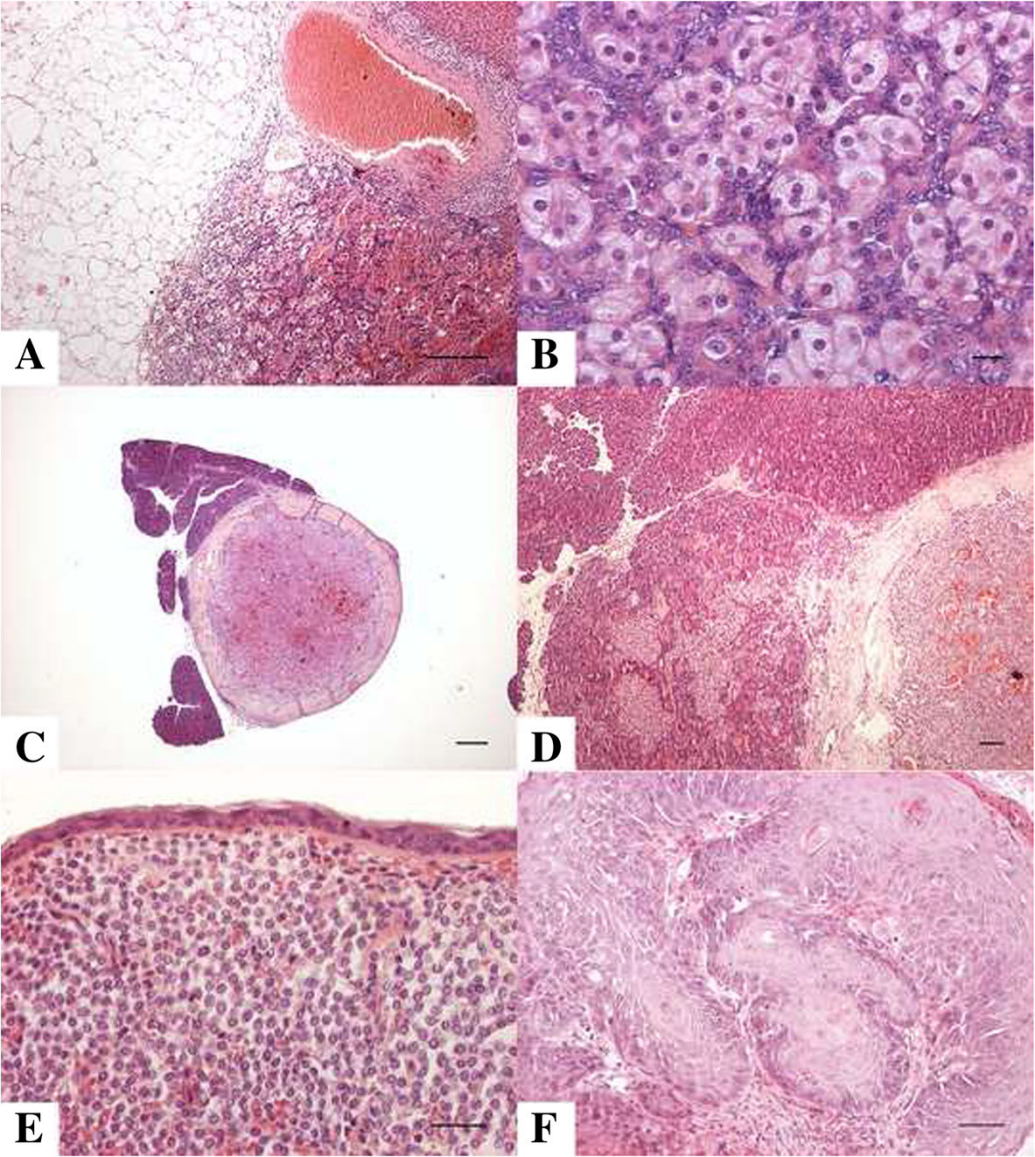
**a** Adrenocortical carcinoma, adrenal gland, ferret. Neoplastic cells infiltrate the capsule and adjacent adipose tissue with a mild desmoplasia, and minimal lymphoplasmacytic inflammation. Bar 50 micron, Hematoxylin and eosin stain. **b** Adrenocortical carcinoma, adrenal gland, ferret. Concurrent presence of large polygonal cells of the zona fasciculate characterized by granular cytoplasm and of smaller cells with high nuclear/cytoplasmic ratio. Bar 50 micron, Hematoxylin and eosin stain. **c** Insular adenoma, pancreas, ferret. Typical expansile growth delimited by a thick fibrous capsule. Cells are organized in dense pockets with minimal highly vascularized stroma. Bar 1 mm, Hematoxylin and eosin stain. **d** Insular adenocarcinoma, pancreas, ferret. The tumor is characterized by infiltration of the capsule and adjacent exocrine pancreas by neoplastic cells. Bar 50 micron, Hematoxylin and eosin stain. **e** Squamous cell carcinoma, skin, ferret. Cords of atypical keratinocytes of the spinous layer are proliferating and invading the superficial dermis. Asynchronous keratinocyte maturation and keratin pearls are visible. Bar 50 micron, Hematoxylin and eosin stain. **f** Well differentiated mast cell tumor, haired skin, ferret. The dermis is diffusely obscured and substituted by dense sheets of well-differentiated mast cells. Bar 50 micron, Hematoxylin and eosin stain

A total of 313 adrenal tumours were diagnosed in 303 ferrets, accounting for 36.6% of cases. Age ranged from 1 to 10 years (mean 4.4, median 4.5 years). Of the 313 adrenal tumours, 289 were cortical (228 carcinomas and 61 adenomas), eight of which were characterized by myxoid differentiation, while 13 derived from the adrenal medulla (11 pheochromocytomas and two neuroblastomas). Ten cases were mesenchymal, including two leiomyosarcomas, five leiomyomas and three spindle cell sarcomas. One case was diagnosed as undifferentiated malignant neoplasm NOS. Mesenchymal tumours were characterized by connection with adrenal gland capsule and compression of adrenal parenchyma. Ten ferrets were affected by two simultaneous neoplasms (six cortical tumours associated with pheochromocytoma, three cortical tumours associated with mesenchymal tumours, and one pheochromocytoma associated with a mesenchymal tumour). The majority of cortical carcinomas were well differentiated and the main criteria for histological malignancy were infiltrative growth in the periadrenal adipose tissue (Fig. 2a and b), invasion of adjacent large vessels and poor demarcation from surrounding tissues [4].

### Pancreas

A total of 200 pancreatic tumours were diagnosed in 196 ferrets, accounting for 23.4% of cases. Age ranged from 2 to 8 years (mean 5.3, median 5 years). Of the 200 pancreatic tumours, 190 were islet cell tumours and ten were exocrine [four adenomas (Fig. 2c) and six adenocarcinomas (Fig. 2d)]. Four ferrets were affected by concomitant insular and pancreatic exocrine tumour. The majority of insular tumours were well differentiated.

### Skin and subcutis

Excluding lymphoid tumours, a total of 156 cutaneous and subcutaneous tumours were diagnosed in 119 ferrets, accounting for 18.2% of the cases. Age ranged from 1 to 10 years (mean 4.7, median 5 years). Sixty-eight mast cell tumours (Fig. 2e) were diagnosed. Sebaceous gland neoplasms were the second most frequent cutaneous neoplasm and included 22 epitheliomas (three of which were associated with a squamous cell carcinoma and five with squamous metaplasia), 16 adenomas (six of which associated with squamous cell carcinoma and three with squamous metaplasia). A total of nine squamous cell carcinomas (Fig. 2f) were diagnosed, accounting for 5.8% of cutaneous tumours, and were all associated with sebaceous tumours.

Eighteen apocrine gland neoplasms were diagnosed (ten adenocarcinomas and eight adenomas). Three out of ten adenocarcinomas were located in the preputial region. Other tumour types included: eight hemangiomas, three fibromas, three leiomyomas, two trichoblastomas, two basal cell carcinomas, one angiosarcoma, one leiomyosarcoma, one rhabdomyosarcoma, one infundibular keratinizing acanthoma, and one lipoma. In this group, 25 ferrets had multiple neoplasms. Of these, sixteen were diagnosed with two tumours, six with three tumours, and three with four tumours.

### Hemolymphatic tumours

Seventy-four tumours affecting the lymphoid tissue were retrieved, accounting for 8.6% of cases. Age ranged from 7 months to 8.5 years (mean 3.4, median 3.5 years). Seventy were lymphomas; the majority were primary nodal (16), 14 splenic, 13 were localized in the gastrointestinal tract (four in the stomach and nine in the intestine), eight were multicentric, five were cutaneous (two of which were epitheliotropic), five hepatic, five hepatosplenic, one pancreatic and one renal. Two cases consisted of an intra-abdominal mass with no connection to recognizable organs or tissues. Four plasma cell tumours were diagnosed: two were splenic, one nodal and one multicentric.

### Skeletal system

Twenty-six tumours arising from the skeletal system were retrieved (3% of the caseload). The majority were chordomas (24 cases) and two were osteosarcomas. Age ranged from 8 months to 7 years (mean 3.7, median 4 years). All chordomas were located on the tail, whereas the two osteosarcomas arose from the maxilla.

### Abdominal cavity

Twenty-four intra-abdominal tumours without connection to any recognizable organ or tissue were diagnosed, accounting for 2.8% of cases. Age ranged from 1 to 8 years (mean 3.4, median 3 years), all ferrets were female. Twenty-two were well demarcated tumours with a diameter ranging from 0.8 to 4 cm, composed mostly of interlacing bundles of spindle cells intermixed with polygonal cells with vacuolated cytoplasm. The remaining two cases were one leiomyosarcoma and one hemangioma respectively.

### Liver

Twenty-six tumours were detected in the liver. Excluding lymphoid tumours, 20 hepatic tumours were diagnosed (3% of the caseload), including six cholangiocellular carcinomas, six vascular tumours (four hemangiosarcomas and two hemangiomas), five hepatocellular tumours (three carcinomas and two adenomas), and three undifferentiated RCTs. Age ranged from 2 to 7 years (mean 4.4, median 4 years).

### Digestive tract

Thirty tumours were detected in the digestive tract. Excluding lymphoid tumours, a total of 15 tumours (accounting for the 1.75% of the caseload) arising from the digestive tract were diagnosed. Age ranged from 2 to 7 years of age (mean 4.2, median 4 years). Five cases were in the oral cavity (three squamous cell carcinomas, one fibromatous epulis and one sarcoma NOS), seven in the intestine (six adenocarcinomas and one leiomyoma) and three were gastric carcinomas.

### Female genital system

Eight tumours, accounting for the 0.9%, were diagnosed. Ovarian neoplasms included three gonadostromal tumours (theca cell tumours) and one cystic papillary adenocarcinoma. Neoplasms arising from the uterus were two adenocarcinomas and one leiomyoma. One single vaginal papilloma was recorded. Age ranged from 1 to 6 years (mean 3.2, median 2.8 years).

### Male genital system

Six tumours were diagnosed (accounting for the 0.7% of the cases) and included three seminomas, two gonadostromal tumours (Leydig cell tumour) and one prostatic adenocarcinoma. Age ranged from 3 to 7 years (mean 5, median 5 years).

### Miscellanea

Other 14 tumours identified, accounting for the 1.6% of the caseload. The miscellanea tumors included: four cases in which a tumour metastatic to the lymph node was the sole sample submitted (three adenocarcinomas with apocrine differentiation and one undifferentiated neuroendocrine tumour), three splenic round cell tumours NOS, one primary nodal hemangioma, one lacrimal gland adenoma, one salivary gland adenoma, one renal sarcoma, one splenic myelolipoma, one mediastinal hemangioma, one pulmonary adenocarcinoma and one sarcoma arising in the thoracic cavity. Age ranged from 3 to 7 years (mean 5.1, median 5 years).

## Discussion

Epidemiological information on tumours in ferrets derives from extra-European countries, mostly USA and Japan [1–4]. In these countries, similar distributions have been reported, with minor discrepancies on the frequency of hemolymphatic and integumentary tumours [1–4]. In our study, the histologic type and tissue distribution of 856 neoplasms from Italian ferrets is reported. A predilection of gender was not observed, and the age of affected ferrets was variable, paralleling the findings of previous reports [1–6].

Additionally, similar to previous reports, endocrine neoplasms represented the majority of tumours in domestic ferrets [1–4]. On the contrary, adrenal tumours were more frequent compared to pancreatic islet cell neoplasms in this report. This discrepancy can be explained partially by the criteria applied in this study to classify islet cell tumours: multiple islet cell tumours in the same sample were regarded as a single case, interpreting the development of multiple identical pancreatic neoplasms in the same animal as the consequence of the same pathological process.

Most adrenal neoplasms were malignant and originated from the cortex, whereas pheochromocytomas were less common, paralleling previous observations [6, 8]. Similar to previous studies [6, 8], no female/male predisposition was highlighted. In fact, early gonadectomy and neutering (commonly performed in Italian ferrets), combined with the artificial prolonged photoperiod experienced by indoor pet ferrets are believed to predispose both genders to the development of adrenocortical tumours.

Interestingly, we observed a higher frequency of mesenchymal neoplasms arising from the adrenal capsule compared with other reports [1–4]. These tumours were characterized by scant amount of residual adrenal tissue and by smooth muscle differentiation in the majority of cases.

Pancreatic islet cells tumours have been well characterized in ferrets over the last 20 years [5], and are reported as the most common neoplasm in middle aged to older ferrets [1–5]. On the contrary, islet cell tumours were the second most common neoplasm in our study, with a majority demonstrating an infiltrative growth. Nevertheless, the infiltrative growth has not been demonstrated to be an indication of malignancy in these tumours [2, 4]. A slight prevalence of male ferrets was observed; this statement is in agreement with previous reports [5].

Cutaneous and subcutaneous mast cell tumours were the most frequent skin neoplasms, followed by sebaceous and apocrine gland tumours. This data parallels one study reporting mast cell tumour as one of the most frequent skin tumour of ferrets [9], but is in contrast with several reports in which sebaceous gland tumours as the most frequent cutaneous neoplasm [1–4].

In our caseload, the number of sebaceous epitheliomas was slightly higher than adenomas. Both tumour types were characterized by squamous differentiation, and by areas of SCC development in nine cases. The presence of squamous metaplasia has been previously reported in these tumours that have been defined as “basal cell tumour with sebaceous and squamous differentiation” and interpreted as putatively arising from pluripotential basal cells [10]. The occurrence of SCC within sebaceous tumours has been previously reported in long-standing lesions in ferrets, but valid hypotheses able to explain this association are lacking [2, 4].

The prevalence of vascular cutaneous/subcutaneous neoplasms in our caseload parallels previous reports [3, 10]. On the contrary, cutaneous neoplasms of smooth muscular origin were less common in this study [3, 11].

The lymphoid tissue was the third most commonly involved by neoplastic disease after endocrine tissues and skin/subcutis. Most lymphomas were nodal, followed by lymphoma arising from the spleen and the gastrointestinal tract. The frequency of lymphoma was lower than what previously reported [1–4]. In the United States, lymphoma has been hypothesized to have a viral etiology in ferrets [12–14]. However, the agent has not been identified even if horizontal transmission of lymphoma using cell or cell free inoculation has been demonstrated, supporting this hypothesis [12–14]. The different frequency of lymphoma in our caseload compared with ferrets from the U.S. could be related to a dissimilar distribution of this putative etiological agent. A further hypothesis may derive from the fact that, in veterinary practice, lymphomas are frequently diagnosed by cytology only; thus, the number of lymphomas in our caseload could be underestimated. Similarly to what is reported in literature, hemangiosarcomas occurred more commonly in the liver, hemangiomas mainly in the skin, and rarely in thoracic and abdominal cavity, likely in association with serosal membranes [4, 15].

Skeletal tumours consisted mainly of chordomas, and were located on the tail as previously reported [3, 16]. On the contrary, osteosarcomas were rare and arose in the maxillary region rather than in the appendicular bones.

It is worth mentioning the high number of spindle cells tumours with vacuolated cells evidenced within the abdominal cavity, without connection to identifiable organs or tissues. No reports of these tumours seem to be available [1–4]. The large size of these neoplasms can be explained by the lack of associated clinical signs, leading to a late diagnosis. In these abdominal tumours, the spindle cell component accounted for almost the 90% of the neoplasm, while the vacuolated component was minimal. The morphology of the polygonal neoplastic vacuolated cells strongly resembled adrenal cortical cells, suggesting a possible origin from ectopic adrenal tissue. The documented presence of a spindle cell component within certain adrenal neoplasms likely supports this hypothesis [17, 18], and has been considered both a normal stromal component of the tumours or part of the tumours itself [17, 18].

## Conclusions

In conclusion, the frequency and the tissue distribution of tumours recorded in this study paralleled previous findings, with some differences regarding the frequency of lymphoma, adrenal mesenchymal tumours and the type of cutaneous tumours. Some tumours that are among the most common in other species, such as hemangiosarcoma, osteosarcoma and SCC, seem to be uncommon in ferrets and to be characterized by distinctive predilection sites. The unusual association between SCC and sebaceous gland neoplasms and the primary origin of intrabdominal spindle cell neoplasms grants further investigation.

## Abbreviations

MCT: Mast cell tumors
NOS: Not otherwise specified
RCT: Round cell tumors
SCC: Squamous cell carcinomas

## References

[1] Li X, Fox JG, Padrid PA (1998). Neoplastic diseases in ferrets: 574 cases (1968-1997). J Am Vet Med Assoc.

[2] Quesenberry KE, Carpenter JW (2012). Ferrets, rabbits, and rodents: clinical medicine and surgery.

[3] Miwa Y, Kurosawa A, Ogawa H, Nakayama H, Sasai H, Sasaki N (2009). Neoplasitic diseases in ferrets in Japan: a questionnaire study for 2000 to 2005. J Vet Med Sci Jpn Soc Vet Sci.

[4] Fox JG, Marini RP (2014). Other systemic diseases. biol. dis. ferret.

[5] Chen S (2008). Pancreatic endocrinopathies in ferrets. Veterinary Clin. North Am. Exot. Anim. Pract.

[6] Simone-Freilicher E (2008). Adrenal gland disease in ferrets. Veterinary Clin. North Am. Exot. Anim. Pract.

[7] Head KW, Armed Forces Institute of Pathology (U.S.), American Registry of Pathology, WHO Collaborating Center for Worldwide Reference on Comparative Oncology (2003). Histological classification of tumors of domestic animals.

[8] Bielinska M, Kiiveri S, Parviainen H, Mannisto S, Heikinheimo M, Wilson DB (2006). Gonadectomy-induced adrenocortical neoplasia in the domestic ferret (Mustela putorius furo) and laboratory mouse. Vet Pathol.

[9] Parker GA, Picut CA (1993). Histopathologic features and post-surgical sequelae of 57 cutaneous neoplasms in ferrets (Mustela putorius furo L.). Vet Pathol.

[10] Kanfer S, Reavill DR (2013). Cutaneous neoplasia in ferrets, rabbits, and guinea pigs. Veterinary Clin North Am Exot Anim Pract.

[11] Mikaelian I, Garner MM (2002). Solitary dermal leiomyosarcomas in 12 ferrets. J Vet Diagn Investig Off Publ Am Assoc Vet Lab Diagn Inc.

[12] Erdman SE, Reimann KA, Moore FM, Kanki PJ, Yu QC, Fox JG (1995). Transmission of a chronic lymphoproliferative syndrome in ferrets. Lab Investig J Tech Methods Pathol.

[13] Erdman SE, Kanki PJ, Moore FM, Brown SA, Kawasaki TA, Mikule KW (1996). Clusters of lymphoma in ferrets. Cancer Invest.

[14] Batchelder MA, Erdman SE, Li X, Fox JG (1996). A cluster of cases of juvenile mediastinal lymphoma in a ferret colony. Lab Anim Sci.

[15] Cross BM (1987). Hepatic vascular neoplasms in a colony of ferrets. Vet Pathol.

[16] Dunn DG, Harris RK, Meis JM, Sweet DE (1991). A histomorphologic and immunohistochemical study of chordoma in twenty ferrets (Mustela putorius furo). Vet Pathol.

[17] Gliatto JM, Alroy J, Schelling SH, Engler SJ, Dayal Y (1995). A light microscopical, ultrastructural and immunohistochemical study of spindle-cell adrenocortical tumours of ferrets. J Comp Pathol.

[18] Newman SJ, Bergman PJ, Williams B, Scase T, Craft D (2004). Characterization of spindle cell component of ferret (Mustela putorius furo) adrenal cortical neoplasms - correlation to clinical parameters and prognosis. Vet Comp Oncol.

